# Amantadine withdrawal syndrome masquerading as COVID-19 encephalopathy: a case report and review of the literature

**DOI:** 10.1093/omcr/omaa133

**Published:** 2021-02-15

**Authors:** John P Murray, Angela Kerins

**Affiliations:** 1 Department of Hospital Medicine, University of Chicago, Chicago, IL, USA; 2 Clinical Pharmacist, University of Chicago, Chicago, IL, USA

## Abstract

Amantadine withdrawal syndrome (AWS) is a rare but recognized cause of severe and persistent altered mental status sometimes with co-occurring extrapyramidal symptoms. First described in a case series from 1987, its clinical manifestations have been characterized along a spectrum ranging from profound hypoactive delirium to hyperactive delirium with hallucinations. Risk factors for withdrawal include abrupt medication discontinuation, prolonged use, older age and underlying dementia.

Herein we describe a case of a 52-year-old woman who presented with confusion, hallucinations, and coronavirus disease-2019 infection. She subsequently developed a prolonged hypoactive delirium after her amantadine was tapered and held. Her hypoactive delirium entirely resolved with resumption of amantadine confirming the diagnosis of AWS. This case illustrates the importance of slowly tapering dopaminergic medications and being aware of rare pharmacologic side effects.

## INTRODUCTION

Amantadine hydrochloride, among the many happy accidents in medicine, was inadvertently discovered to alleviate the extrapyramidal symptoms of Parkinson’s disease (PD) in 1968. A woman with PD found her symptoms much improved while taking amantadine for flu prophylaxis. Later that year a large trial demonstrated both subjective and objective benefits when used to treat PD [1]. Since that time, amantadine has been used to treat PD alongside a number of other movement disorders.

Although its mechanism is not fully understood, amantadine is thought to achieve its effect via N-methyl-D-aspartate receptor antagonism, direct and indirect effects on dopamine neurons, and by decreasing anticholinergic tone [2]. Amantadine can cause dopamine toxicity: paranoia, hallucinations and tachycardia. For this reason, the medication is initiated progressively. Inversely, if it is rapidly discontinued, patients may develop amantadine withdrawal syndrome (AWS), a severe and persistent delirium often with concurrent extrapyramidal symptoms.

## CASE REPORT

A 52-year-old female with a past medical history of spinocerebellar ataxia, hyperthyroidism and depression presented to the hospital with 2–3 weeks of hallucinations. Admission labs were remarkable for a new acute kidney injury with blood urea nitrogen (76 mg/dl) and creatinine (1.6 mg/dl from baseline of 0.8 mg/dl). Additionally, she was found to be coronavirus disease-2019 (COVID-19) positive. Complete blood count, urinalysis, thyroid stimulating hormone (TSH), serum ethanol level and head computed tomography were unremarkable. Vital signs were notable for a heart rate of 104. On arrival she was alert and answering questions appropriately. The patient had been prescribed amantadine for 2.5 years prior to admission and was taking 300 mg daily.

The neurology service suspected her hallucinations were related to amantadine toxicity and recommended a 3-day taper, shortened to 2 days by the primary service. Serum amantadine level was ordered on admission and found to be 1505 ng/dl (therapeutic range 200–1000 ng/ml and toxicity >2000 ng/ml). The patient continued to have hallucinations with concurrent agitation, prompting an electroencephalogram (EEG) and lumbar puncture. All lumbar puncture studies were normal. EEG showed background slowing consistent with encephalopathy with no epileptiform activity.

After 3 days, the patient was no longer having active hallucinations but became increasingly somnolent and disoriented. Five days into her course, she was no longer consistently speaking with providers. Two weeks into her hospitalization, she required assistance in feeding and was not reliably following commands. Given her persistent symptoms, the possibility of COVID-19 encephalopathy was raised and a repeat lumbar puncture considered for colony-stimulating factor polymerase chain reactiontesting. Prior to this, the patient was resumed on 200 mg of oral amantadine. Within 2 days she was alert, speaking in full sentences, and oriented to person, month and situation. Because the patient’s prolonged delirium resolved immediately after resuming amantadine, the diagnosis of AWS was made. The neurology service believed that the patient’s initial presentation was consistent with amantadine toxicity with the patient going into withdrawal in the subsequent days as the medication was tapered and held, thus explaining both her initial agitation and hallucinations and subsequent hypoactive delirium. She ultimately returned to her cognitive baseline and was discharged to subacute rehab.

## DISCUSSION

Since 1987 there have been seven case reports describing instances of AWS including 15 patients. This is the first published since 2017 and is noteworthy in that it describes a patient without PD or underlying dementia. The first case series, published by Wilson in 1987, describes three older patients who experienced recrudescence of their PD motor symptoms after amantadine was held [3]. Only one of the three patients may have suffered from our more contemporary definition of AWS, an acute delirium following the discontinuation of the medication, whereas the other two patients experienced only movement symptoms.

In 1997 Factor published a case series describing three elderly patients who developed acute delirium with worsening motor function after amantadine was held [4]. All patients had underlying dementia and had been taking amantadine for more than 4 years. Symptoms quickly resolved after resuming the medication. Interestingly, all patients had their amantadine held due to hallucinations. Miyasaki republished a reply to the editor sharing two similar cases of patients with longstanding PD who developed AWS [5]. Factor responded with two further cases of elderly patients with characteristic PD and AWS. These cases were novel in that one patient did not have underlying dementia and both patients had been taking amantadine for less than 1 year. Again, symptoms entirely resolved with resumption of amantadine. Miyasaki hypothesized that pathogenesis of the delirium was related to the glutamatergic system. At this point some general risk factors for AWS were induced: old age, advanced PD, underlying dementia and duration of therapy. Factor agreed with Miyaski that AWS was unlikely related to dopaminergic pathways.

In 2009 Brantley published a case report of neuroleptic malignant syndrome (NMS) believed induced by amantadine withdrawal [6]. The authors hypothesized that more classic AWS exists on a spectrum which includes NMS. Marxreiter added an additional case of severe AWS in a patient with PD in 2017, helpfully noting the benefit of reintroducing amantadine early in instances of diagnostic uncertainty due to its rapid effects [7]. Finally, Fryml described three cases of AWS in a 2017 report which interestingly included the first patient without PD [8]. The authors noted the remarkable duration that AWS can persist: in one instance for weeks. Similar to Brantley, she describes AWS as a protean syndrome ranging from delirium to NMS, driven by dopaminergic derangements.

Our case illustrates both characteristic and unusual features of AWS. Characteristically, this case was prolonged and refractory, included motor symptoms, and entirely resolved with amantadine reintroduction. Atypically, this case involved a patient without PD who experienced a hypoactive delirium, although this has been also reported. Intriguingly, this case of AWS likely occurred after a period of amantadine toxicity, as the patient initially presented with hallucinations and elevated serum amantadine levels. We hope this report highlights the importance of prolonged amantadine tapers in the setting of toxicity as symptoms of overdose and withdrawal can overlap, creating diagnostic confusion. Further questions which remain to be answered include the exact pathophysiology of the syndrome and if it includes NMS as part of its spectrum.
